# Supplementary material for: Novel Driver Strength Index highlights important cancer genes in TCGA PanCanAtlas patients
Source: PeerJ. 2022 Aug 11;10:e13860. doi: 10.7717/peerj.13860 (PMC9375969; doi:10.7717/peerj.13860)
Supplement: Supplemental Information 2 [file peerj-10-13860-s002.zip › COHORTS/genes plots/detailed/2021_8_16_14_9_distribution_events_detailed_males_5.pdf]

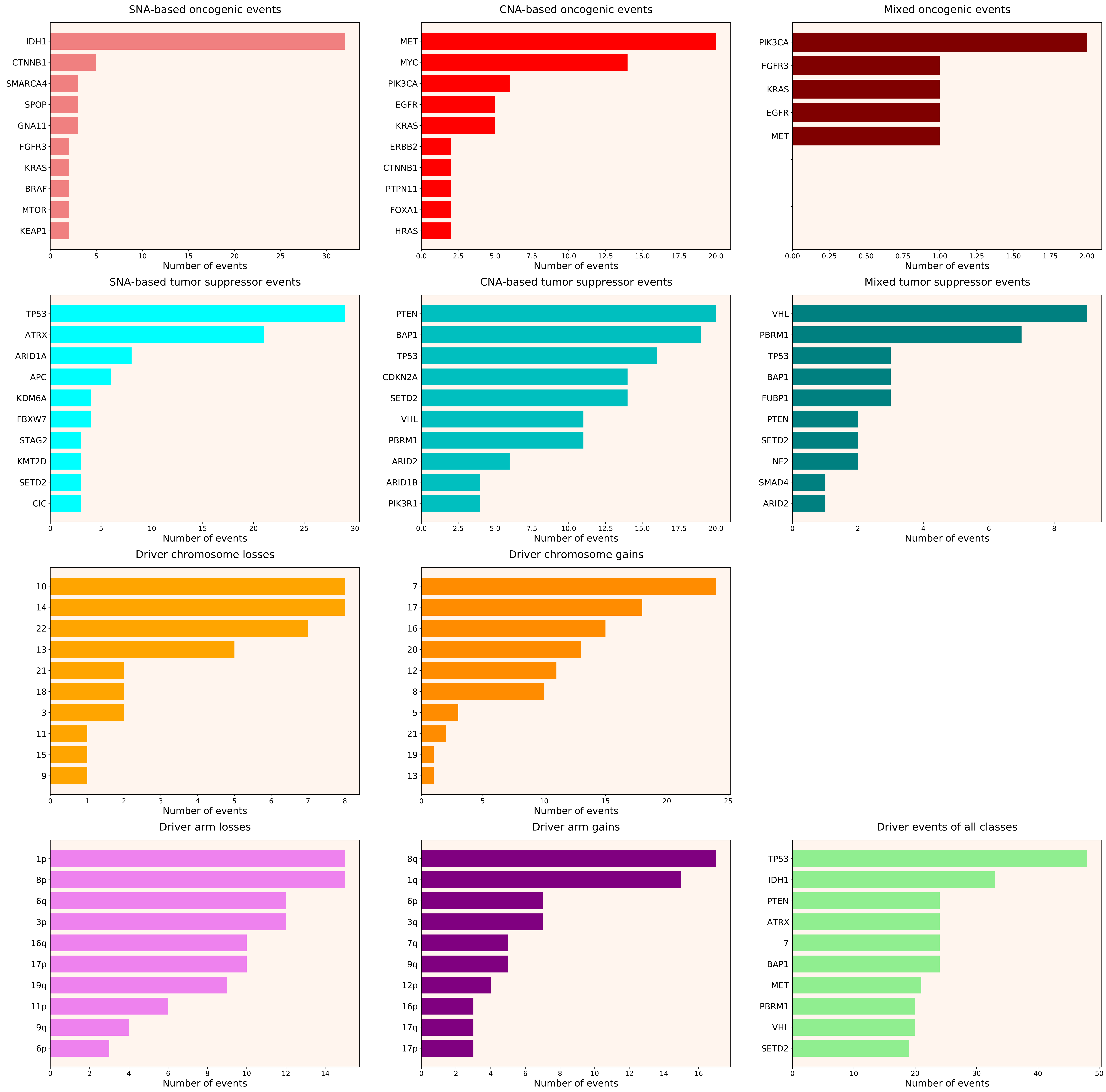

SNA-based oncogenic events
IDH1
CTNNB1
SMARCA4
SPOP
GNA11
FGFR3
KRAS
BRAF
MTOR
KEAP1
Number of events
CNA-based oncogenic events
MET
MYC
PIK3CA
EGFR
KRAS
ERBB2
CTNNB1
PTPN11
FOXA1
HRAS
Number of events
Mixed oncogenic events
PIK3CA
FGFR3
KRAS
EGFR
MET
Number of events
SNA-based tumor suppressor events
TP53
ATRX
ARID1A
APC
KDM6A
FBXW7
STAG2
KMT2D
SETD2
CIC
Number of events
CNA-based tumor suppressor events
PTEN
BAP1
TP53
CDKN2A
SETD2
VHL
PBRM1
ARID2
ARID1B
PIK3R1
Number of events
Mixed tumor suppressor events
VHL
PBRM1
TP53
BAP1
FUBP1
PTEN
SETD2
NF2
SMAD4
ARID2
Number of events
Driver chromosome losses
10
14
22
13
21
18
3
11
15
9
Number of events
Driver chromosome gains
7
17
16
20
12
8
5
21
19
13
Number of events
Driver arm losses
1p
8p
6q
3p
16q
17p
19q
11p
9q
6p
Number of events
Driver arm gains
8q
1q
6p
3q
7q
9q
12p
16p
17q
17p
Number of events
Driver events of all classes
TP53
IDH1
PTEN
ATRX
7
BAP1
MET
PBRM1
VHL
SETD2
Number of events
